# Synergistic Disruption of External Male Sex Organ Development by a Mixture of Four Antiandrogens

**DOI:** 10.1289/ehp.0900689

**Published:** 2009-07-15

**Authors:** Sofie Christiansen, Martin Scholze, Majken Dalgaard, Anne Marie Vinggaard, Marta Axelstad, Andreas Kortenkamp, Ulla Hass

**Affiliations:** 1 Department of Toxicology and Risk Assessment, National Food Institute, Technical University of Denmark, Søborg, Denmark; 2 School of Pharmacy, University of London, London, United Kingdom

**Keywords:** antiandrogens, azole fungicides, combination effects, cumulative effects, DEHP, dose addition, finasteride, independent action, male sexual differentiation, mixtures, phthalates, prochloraz, vinclozolin

## Abstract

**Background:**

By disrupting the action of androgens during gestation, certain chemicals present in food, consumer products, and the environment can induce irreversible demasculinization and malformations of sex organs among male offspring. However, the consequences of simultaneous exposure to such chemicals are not well described, especially when they exert their actions by differing molecular mechanisms.

**Objectives:**

To fill this gap, we investigated the effects of mixtures of a widely used plasticizer, di(2-ethylhexyl) phthalate (DEHP); two fungicides present in food, vinclozolin and prochloraz; and a pharmaceutical, finasteride, on landmarks of male sexual development in the rat, including changes in anogenital distance (AGD), retained nipples, sex organ weights, and malformations of genitalia. These chemicals were chosen because they disrupt androgen action with differing mechanisms of action.

**Results:**

Strikingly, the effect of combined exposure to the selected chemicals on malformations of external sex organs was synergistic, and the observed responses were greater than would be predicted from the toxicities of the individual chemicals. In relation to other hallmarks of disrupted male sexual development, including changes in AGD, retained nipples, and sex organ weights, the combined effects were dose additive. When the four chemicals were combined at doses equal to no observed adverse effect levels estimated for nipple retention, significant reductions in AGD were observed in male offspring.

**Conclusions:**

Because unhindered androgen action is essential for human male development in fetal life, these findings are highly relevant to human risk assessment. Evaluations that ignore the possibility of combination effects may lead to considerable underestimations of risks associated with exposures to chemicals that disrupt male sexual differentiation.

Certain chemicals present in consumer products and food can disrupt the action of steroidal androgens in fetal life, with irreversible consequences for later life stages. In the male rat, exposure to such chemicals during development leads to incomplete masculinization and severe malformations of the reproductive organs (Christiansen et al. 2008; Welsh et al. 2008; Wilson et al. 2008). Because of their relevance to humans, the effects seen in laboratory animals have led to considerable concerns. Substances of interest, so-called antiandrogens, include certain phthalates (widely used as plasticizers), a few pharmaceutical agents, and some fungicides. Phthalates have attracted significant recent interest, with several regulatory bodies currently engaged in risk assessments. As yet, these regulatory efforts have taken little account of realistic human exposures to several phthalates and other antiandrogens simultaneously (Blount et al. 2000; Brock et al. 2002; Main et al. 2006; Marsee et al. 2006; Swan et al. 2005). With the aim of exploring the effects of combined exposure to antiandrogens during gestation, we conducted large developmental toxicity mixture experiments in the rat with the widely used plasticizer di(2-ethylhexyl) phthalate (DEHP), the fungicides vinclozolin and prochloraz (present in certain food items), and the pharmaceutical finasteride.

We chose to examine these antiandrogens because they can disrupt male sexual differentiation in different ways, by a variety of mechanisms. In fetal life, testosterone is a key driver of the differentiation of the Wolffian duct system into the vas deferens, epididymis, seminal vesicles, and external genitalia. Phthalates with a certain ester side-chain length, such as DEHP, can lower testosterone levels by interfering with the uptake of cholesterol precursors into fetal Leydig cells, where testicular androgen production takes place (Foster et al. 1980). In the rat, malformations of internal reproductive organs (epididymis, testes) are the consequence. Because dihydrotestosterone (DHT) is derived from testosterone through enzymatic conversion by 5α-reductase, lower testosterone concentrations also affect the development of tissues that rely on DHT (prostate and external genitalia). DHT is further required for the regression of nipple anlagen in male rats and for the growth of the perineum to produce the normal male anogenital distance (AGD), which is longer than in females (Imperato-McGinley et al. 1985, 1986). Because of reduced DHT levels in the wake of suppressed testosterone synthesis, retained nipples and feminized AGDs are also seen in male rats exposed to phthalates in fetal life (Carruthers and Foster 2005; Foster 2006; Jarfelt et al. 2005). Vinclozolin metabolites affect more directly the development of DHT-dependent tissues by antagonizing the androgen receptor (AR) (Gray et al. 1999; Hass et al. 2007; Wolf et al. 2000). In disrupting the enzymatic conversion of testosterone to DHT through inhibition of 5α-reductase (Bowman et al. 2003; Clark et al. 1990), the pharmaceutical finasteride induces an effect spectrum similar to that of AR antagonists. Finally, the fungicide prochloraz disrupts androgen action by inhibiting the conversion of progesterone to testosterone and by antagonizing the AR (Blystone et al. 2007; Laier et al. 2006; Noriega et al. 2005; Vinggaard et al. 2002, 2005).

One goal of mixture toxicology is to anticipate the toxicities of untested mixtures of chemicals when only the effects of individual components are known (Hermens et al. 1984, 1985; Könemann 1980, 1981). A second issue of relevance to regulatory toxicology concerns the question of mixture effects at low doses. A view that has persisted over the last 30 years is that combination effects do not occur when each chemical is present at doses equal to their no observed adverse effect levels (NOAELs) or lower [Committee on the Toxicity of Chemicals in Food, Consumer Products and the Environment (COT) 2002; Norwegian Scientific Committee for Food Safety (VKM) 2008]. This is thought to be the case with chemicals that act through different mechanisms, but not with agents that target similar molecular structures. The NOAEL is the highest tested dose at which no statistically or biologically adverse effects can be identified, and is used in regulatory toxicology as a point of departure for establishing “acceptable” exposures for humans.

In experiments with rats, the combined effects of antiandrogens that act through similar mechanisms, such as several phthalates that suppress testosterone synthesis (Howdeshell et al. 2008) or some AR antagonists (Christiansen et al. 2008; Hass et al. 2007; Metzdorff et al. 2007), were additive and could be predicted well from the effects of the individual mixture components. In addition, combination effects were observed at NOAELs for the individual chemicals. Less obvious is how combinations of antiandrogens behave to disrupt androgen action via different mechanisms. In the present study we investigated the predictability of the combined effects of mixtures composed of dissimilarly acting antiandrogens. In pursuing this aim, we decided to combine all chemicals in the mixture in proportion to their NOAELs for effects on male sexual development. This was done in order to investigate whether combination effects occur at individual doses of the compounds that are of regulatory relevance, and in order to avoid the testing situation in which one or two chemicals contribute disproportionately to an overall combination effect. Our motivation was not to model environmental intakes. A second goal was to test whether combination effects occur at doses around the NOAELs for the single chemicals; this also forced us to combine the antiandrogens in proportion to their relative potency rather than their environmental exposures.

## Materials and Methods

### Chemicals

We obtained vinclozolin (CAS no. 50471-44-8; purity 99%), finasteride (CAS no. 98319-26-7; purity 99.8%), DEHP (CAS no. 117-81-7; purity 99%), and prochloraz (CAS no. 67747; purity 99.6%) from VWR-Bie & Berntsen (Herlev, Denmark). The chemicals were dissolved in corn oil (used as vehicle) from VWR-Bie & Berntsen.

### Animals and dosing

Time-mated nulliparous, young adult Wistar rats [HanTac:WH; Taconic Europe, Ejby, Denmark; body weight (bw) ~ 200 g] were supplied at day 3 of pregnancy. The day after mating was designated gestational day (GD) 1, and postnatal day (PND) 0 was the day of birth. On the day after arrival at our facilities (GD4), the dams were randomly distributed into groups of 16 or 8 animals with similar distributions. A complete rodent diet for growing animals (Altromin 1314; soy- and alfalfa-free; Altromin GmbH, Lage, Germany) and acidified tap water (to prevent microbial growth) was provided *ad libitum*.

**Figure 1:**
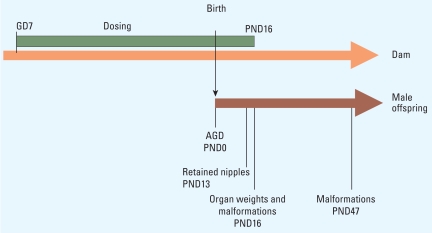
Outline of the study design adopted for dose–response analyses of antiandrogens and their mixtures. Mated dams were dosed from GD7 to PND16. Male offspring were examined for multiple signs of disrupted sexual development, including AGD on PND0, retained nipples (PND13), weights of prostate and LABC (PND16), and malformations of the genital organs (PND16, PND47).

Test chemicals and mixtures were administered to the dams by gavage from GD7 to the day before expected birth (GD21) and from PND1 until PND16 (Figure 1), in a dosing volume of 2 mL/kg bw. The dose levels and group sizes are shown in Table 1. We performed the studies using an experimental randomized design of 4 blocks (with 1 week between) and implemented in each block the same dose regime and number of dams.

### Studies and dose levels

Before conducting the mixture experiment, we performed dose–response studies for each individual chemical. The dose ranges were chosen with the aim of covering the entire range of responses, from no apparent effects to complete feminization, as determined by measurements of AGD and nipple retention (NR). The same approach was used in our first mixture study (Hass et al. 2007). The dose levels selected for the dose–response studies were based on the reductions of AGD and increases of NR reported for vinclozolin (Gray et al. 1994, 1999; Hellwig et al. 2000; Hotchkiss et al. 2002; Shimamura et al. 2002), finasteride (Bowman et al. 2003; Clark et al. 1990; Hib and Ponzio 1995), DEHP (Gray et al. 2000; Jarfelt et al. 2005; Wolf et al. 1999), and prochloraz (Laier et al. 2006). To gain information about the variability of effects between studies, as well as to facilitate a direct comparison within the mixture study, selected doses of vinclozolin, finasteride, DEHP, and prochloraz were run in parallel with the mixture experiment.

Two or three selected doses of the four chemicals were administered in parallel with the mixture doses. For the mixture experiment, a master mixture was prepared by combining doses of the four chemicals at a fixed ratio of 500:1:300:500 (vinclozolin: finasteride:DEHP:prochloraz). Thus, the master mixture contained 15,000 mg vinclozolin, 30.0 mg finasteride, 9,000 mg DEHP, and 15,000 mg prochloraz in 600 mL corn oil. The master mixture was administered undiluted in a dosing volume of 2 mL/kg bw to achieve the highest mixture dose of 130.1 mg/kg/day. Dilutions of 1:1 and 1:9 in corn oil were prepared for the two remaining dose groups of 65.05 mg/kg/day and 13.01 mg/kg/day, respectively. The lowest mixture dose was equivalent to the sum of the chemicals’ individual NOAELs for retained nipples (NR NOAEL), the middle dose was 5-fold higher, and the highest dose was 10-fold this value. The animals were treated humanely and with regard for alleviation of suffering. The animal studies were performed under conditions approved by the Danish Agency for Protection of Experimental Animals and by the in-house Animal Welfare Committee of the National Food Institute at the Technical University of Denmark.

### Determination of changes in male sexual differentiation

#### AGD and NR

In all studies, AGD and NR were recorded by the same technician who was blinded with respect to exposure groups. After birth (PND0), all live pups in the litter were weighed and sexed, and AGD was measured using a stereomicroscope. We transformed the crude AGD measurements to the AGD index by dividing AGD by the cube root of body weight. The cube root was used because this method allowed us to convert a three-dimensional end point (weight) into a one-dimensional end point (AGD) (Gallavan et al. 1999; Wolf et al. 1999). Using this ratio required us to assume that the relationship between AGD and transformed body weight is directly proportional and linear. To assess the validity of this assumption, we explored the influence of using the transformed body weight as a covariable in statistical analyses. However, we found no relevant differences between these two approaches. In the interest of keeping the model parameters as simple as possible (to avoid overparameterization), we decided to base all statistical analyses on the AGD index.

The body weights of all pups were recorded on PND13, together with the number of nipples/areolas, defined as a dark focal area (with or without a nipple bud) located where nipples are normally present in female offspring. Females normally have 12 nipples, but may in a few cases have up to 14.

#### Organ weights and assessment of malformations in external genitalia

On PND16, two to six male pups per litter were weighed and their external genitalia were inspected. From one male per litter, we excised and weighed the following organs: testis, epididymis, ventral prostate, seminal vesicles, levator ani/bulbocavernosus muscles (LABC), bulbourethral glands, adrenals, kidney, and liver. In all examined males, the level of demasculinization was scored on a scale from 0 to 3, with the observer being blinded with respect to dose group. The scores were as follows:

- Score 0 (no effect): The genital tubercle was normal, with the urethral opening at the tip of the genital tubercle and the preputial skin intact. In the perineal area, thick fur extended caudally from the base of the genital tubercle and half the distance to the anus. A furless area circumscribed the anus.
- Score 1 (mild dysgenesis of the external genitals): A small cavity on the inferior side of the genital tubercle or a minor cleft in the preputial opening was observed (estimated value of 0.5–1.4 on an arbitrary scale). The size of the genital tubercle was decreased. The furless area around the anus expanded toward the base of the genital tubercle, but thick fur was still present at the base of the genital tubercle.
- Score 2 (moderate dysgenesis of the external genitals): The preputial cleft was larger (estimated value of 1.5–2.4 on an arbitrary scale). The urethral opening was situated halfway down toward the base of the genital tubercle (hypospadias). Partly furless areas or thin fur was noted in the perineal area extending from the base of the genital tubercle and caudally to the furless area circumscribing the anus.
- Score 3 (severe dysgenesis of the external genitals): The preputial cleft was large (estimated value of 2.5–3.5 on an arbitrary scale). The urethral opening was situated farther than halfway down the inferior side of the genital tubercle to the base of the genital tubercle (hypospadias). At the base of the genital tubercle, a groove extended laterally (similar to females on PND16). The male was totally furless in the entire perineal area.

For data analysis, we simplified the scoring into a binary variable, with scores 0 and 1 (no cleft phallus) versus scores 2 and 3 (clear and marked cleft phallus).

One male per litter was held after weaning in order to examine the external reproductive organs after sexual maturation. On PND47, malformations and changes of the external male reproductive organs, including cleft phallus/hypospadias and blind vaginal opening, were scored by the same technician, again blinded with respect to exposure groups. The changes were scored on a scale from 0 to 3 using the following criteria: 0, normal external reproductive organs; 1, slight cleft or variations of preputium but no hypospadias or blind vaginal opening; 2, clear hypospadias but no blind vaginal opening; 3, marked hypospadias with visible os penis and blind vaginal opening. For data analysis, we transformed the scoring into a binary variable, with scores 0 and 1 (no hypospadias) versus scores 2 and 3 (clear and marked hypospadias).

### Data normalizations

To enable pooling of the data from our studies, which in some cases stretched over a period of 3 years, all regression analyses had to be based on normalized values. For organ weight data from PND16, we found a linear relationship between organ weights and body weight, based on all control pups, with the corresponding regression lines going through the origin within their 95% confidence belts. Therefore, individual organ weights were normalized to the ratio between that organ weight and body weight. Furthermore, we controlled for slight differences in control values between studies by standardizing these ratios to 1 by dividing them through the mean normalized organ weight from unexposed male pups. In a similar way, we standardized the absolute AGD indices to relative values between 1 (no effect on male AGD index) and 0 (complete feminization). The mean AGD indices from unexposed male and female pups were used to define the minimum and maximum responses, respectively. Assessment of NR yielded count values, which were used without further normalization. Data normalizations were also not necessary in the case of the binary malformation scores.

### Dose–response analyses

By using Shapiro-Wilk’s and Bartlett’s tests, we confirmed that all continuous effect measurements were normally distributed and homogeneous. Responses different from controls were assessed for statistical significance by multiple testing methods (global error rate α = 5%, two-sided). Statistical analyses were always adjusted for litter effects by using litter as an independent, random, and nested factor; statistical significance was then assessed on the basis of multiple contrast tests (for additional details, see Hass et al. 2007). When organ weights were analyzed, body weight was included as covariate.

Statistical dose–response regression analyses were conducted in the same way for the mixture and the single compounds, with analyses for the single compounds based on pooled effect data from initial dose–response studies and the repeated doses that were run concurrently with the mixture study [see Supplemental Material, Tables 1 and 2, available online (doi:10.1289/ehp.0900689.S1 via http://dx.doi.org/)]. We used a best-fit approach (Scholze et al. 2001), where various nonlinear regression models (logit, probit Weibull, generalized logit) were fitted independently to the same data set. The best-fitting model was selected on the basis of a statistical goodness-of-fit criterion [Schwarz information criterion (Schwarz 1978)]. To control for litter effects, dose–response data were analyzed by using a generalized nonlinear mixed modeling approach (Vonesh and Chinchilli 1996), with litter as a random effect modifier for individual effect data. The dose–response functions described by Scholze et al. (2001) assume monotonic increasing relationships, but some end points studied here yielded monotonic decreasing forms. To maintain compatibility with the dose–response functions described by Scholze et al. (2001), we exchanged the upper and lower asymptotes in the regression models; that is, the θ_max_ values in the corresponding functions of Table 2 were used as θ_min_, and vice versa.

All analyses were carried out using the SAS procedures PROC GENMOD, PROC MIXED, and PROC MULTTEST (SAS version 8; SAS Institute Inc., Cary, NC, USA).Additional details are published elsewhere (Hass et al. 2007; Metzdorff et al. 2007; Christiansen et al. 2008).

### Calculation of mixture-effect predictions

In making predictions for the combined effects of DEHP, vinclozolin, prochloraz, and finasteride, we assumed that each chemical in the mixture did not exacerbate or diminish the effects of the other components. Mixture effects according to such “no interaction” or “additivity” assumptions can be calculated using two alternative concepts, dose addition and independent action. Dose addition looks at mixture effects in terms of a “dilution principle.” It assumes that one chemical can be replaced totally or in part by an equal fraction of an equieffective dose of another, without diminishing the overall combined effect (Loewe and Muischnek 1926). Dose addition is often used for mixtures composed of chemicals that act through a similar or common mode of action [COT 2002; U.S. Environmental Protection Agency (EPA) 1986, 2000, 2002]. Its application to the present mixture of four antiandrogens appeared justified because all chemicals affect the biological action of testosterone and DHT during development and produce common effect outcomes. However, it can be argued with equal justification that the similarity assumption characteristic for dose addition is not applicable to the chosen mixture because its component chemicals produce their effects by a variety of molecular mechanisms. For this reason, we also employed the alternative concept of independent action to construct additivity expectations. Independent action assumes that the joint effects of a combination of agents can be calculated by adopting the statistical concept of independent events (Bliss 1939). It is viewed as appropriate for mixtures of chemicals with diverse modes of action (COT 2002); however, dose addition and independent action can yield very similar additive mixture-effect predictions at low doses.

Under the assumption of dose-additive combination effects, or those conforming with independent action, we predicted dose–response relationships for the mixture with a defined mixture ratio (“fixed mixture ratio design”; Faust et al. 2001) using the best-fit dose–response regression curves of the individual compounds [see Supplemental Material, Tables 1–3 (doi:10.1289/ehp.0900689.S1)] and compared them with the observed mixture effects (Supplemental Material, Table 3). Equation 1 allows the calculation of any effect dose of a mixture under the hypothesis of dose additivity. For this, the dose–response functions of all mixture components and the mixture ratios have to be known:


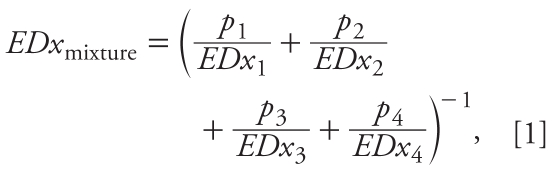


where *EDx*_1_, *EDx*_2_, *EDx*_3_, and *EDx*_4_ are the effect doses of vinclozolin, finasteride, DEHP, and prochloraz, respectively, that on their own produce the same quantitative effect *x* as the mixture, and *p*_1_, *p*_2_, *p*_3_, and *p*_4_ are the relative proportions of the corresponding individual doses present in the total mixture dose. We derived the individual effect doses from the dose–response functions for the compounds by using their inverse functional form. If no effects were observed in the tested dose ranges for the *i*th compound, we replaced the missing information about the effect doses in Equation 1 (*EDx_i_*) by assuming either a maximal effect (realized mathematically by approximating a “yes/no” step function through a dose–response logit function with model steepness parameter θ_2_ = 40) or no effect at all (i.e., setting the ratio *p_i_*/*EDx_i_* in Equation 1 to zero) for doses exceeding those that were actually tested. This procedure had to be adopted in order to calculate mixture-effect predictions for genital malformations, because two of the mixture components, DEHP and prochloraz, were without observable effects on their own [see Supplemental Material, Tables 1 and 3 (doi:10.1289/ehp.0900689.S1)]. These two extrapolation scenarios define the only possible prediction range of mixture responses according to Equation 1.

The basic version of independent action has been formulated under the simple assumption that the susceptibilities of the individuals of an at-risk-population to different dissimilarly acting mixture compounds are not correlated with each other (Bliss 1939). For a four-component mixture this can be defined by


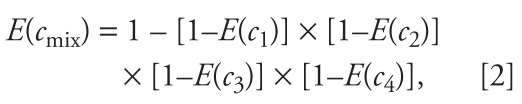


where *E*(*c*_1_), *E*(*c*_2_), *E*(*c*_3_), and *E*(*c*_4_) denote the fractional effects (*x*%) caused by the individual concentrations *c*_1_, *c*_2_, *c*_3_, and *c*_4_ of vinclozolin, finasteride, DEHP, and prochloraz, respectively, and *E*(*c*_mix_) is the total effect of the mixture concentration *c*_mix_. The individual effects of mixture compounds *E*(*c_i_*) are estimated from the concentration response functions determined for single substances (Table 1). Equation 2 cannot be transformed into an explicit term for an effect mixture dose *EDx*_mix_, but the value of *EDx*_mix_ satisfying Equation 2 for a given effect level *x* has to be computed by an iterative procedure. The statistical uncertainty for the predicted mixture effects and effect doses was determined using the bootstrap method (Efron and Tibshirani 1993) and expressed as 95% confidence limits for the predicted mean estimate. Differences between predicted and observed effect doses were deemed statistically significant when the 95% confidence belts of the prediction did not overlap with those of the experimentally observed mixture effects.

## Results

### Dose–response analyses with the individual mixture components

Of all indicators of disrupted sexual development, alterations in retained nipples were the most sensitive end point, with effects becoming noticeable at the lowest doses. Changes in AGD were almost as sensitive, followed by reductions in prostate, LABC weights, and genital malformations. Finasteride was by far the most potent chemical. It exhibited dose–response curves with very shallow gradients for all end points. Vinclozolin was active in a narrower dose range, with correspondingly steeper dose–response curves, and the characteristics of prochloraz and DEHP fell between these extremes. Details of the dosing schedules and the timing of the various studies are presented in Table 1. A summary of dose–response descriptors for all tested chemicals, based on the pooled outcome of studies conducted before the mixture experiment, is available in Supplemental Material, Tables 1–3 (doi:10.1289/ehp.0900689.S1); responses seen with individual chemicals tested in parallel with the mixture are described in Supplemental Material, Table 4.

### Prediction of mixture effects

The mixture ratio used in our study was chosen to reflect the NOAELs of each chemical alone, judged in relation to the most sensitive indicator of disruption of male sexual development, increased NR. These NOAELs, here referred to as NR NOAELs, were estimated in our laboratory before the mixture experiment began (3 mg/kg/day for DEHP, 5 mg/kg/day for vinclozolin, 5 mg/kg/day for prochloraz, and 0.01 mg/kg/day for finasteride). Our NR NOAELs agree well with the doses that are currently used as starting points for establishing tolerable exposures for humans [see Supplemental Material, Table 5 (doi:10.1289/ehp.0900689.S1)]. We tested mixture doses equal to the sum of NR NOAELs (13.01 mg/kg/day) and multiples of 5 (65.05 mg/kg/day) and 10 (130.1 mg/kg/day).

### Additive mixture effects

**Figure 2:**
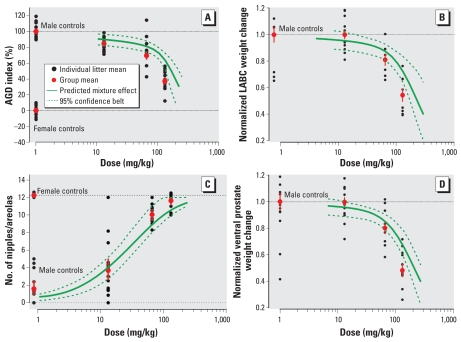
Prediction and assessment of combination effects of antiandrogens with mixed modes of action on hallmarks of male sexual development in rats exposed gestationally to a combination of DEHP, vinclozolin, prochloraz, and finasteride with a mixture ratio of 3:5:5:0.01. Doses shown are total mixture doses. (*A*) Changes in anogenital index at PND1. (*B*) Weight changes of the LABC at PND16 normalized in relation to body weight and untreated controls. (*C*) Number of retained nipples at PND13. (*D*) Weight changes of ventral prostates at PND16 normalized to body weight and untreated controls. The predicted mixture effects were derived by using dose addition. Error bars for group means indicate SEM.

Figure 2 shows how the experimentally observed responses compared with the additive effects of the mixture that were predicted using dose addition. The effect outcomes of the mixture experiment are available in Supplemental Material, Table 4 (doi:10.1289/ehp.0900689.S1). Changes in AGD, retained nipples, prostate weights, and weights of LABC were chosen as the end points for evaluation. In all cases, the overall effect means measured for all litters came close to the predicted response curves. We found considerable overlap between the mean responses for individual litters and the anticipated effects. To evaluate the relationship between predictions and observations statistically, we used the effect variability seen with the single chemicals to estimate statistical confidence limits for the predicted (dose-additive) effect doses by applying bootstrapping methodology. A statistically significant difference between anticipated and observed effect dose could not be detected, except for doses that caused changes in AGD at an effect level of 60% change relative to controls (Table 2). The mixture-effect doses predicted using the alternative concept of independent action did not differ significantly from those anticipated by dose addition. For all end points, the observed mixture effects were stronger than the responses attributable to any one single mixture component. Our data suggest that the combined effects of DEHP, vinclozolin, prochloraz, and finasteride are additive when the evaluation is based on changes in AGD, retained nipples, prostate weights, and weights of LABC.

### Synergistic effects for genital malformations

**Figure 3:**
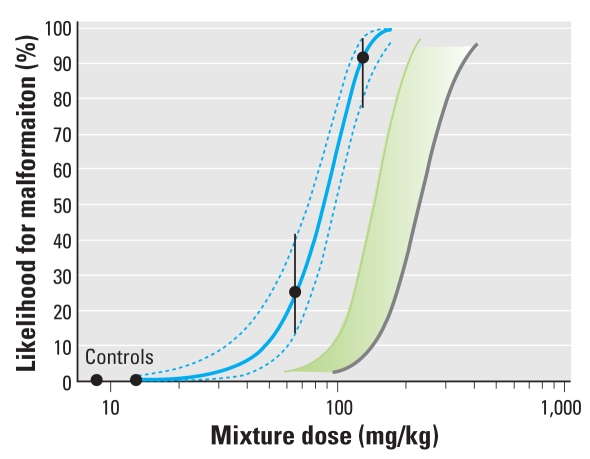
Synergistic effects on induction of genital malformations on PND16 in male rats exposed gestationally to combinations of DEHP, vinclozolin, prochloraz, and finasteride with a mixture ratio of 3:5:5:0.01. Doses shown are total mixture doses. Genital malformations include enlarged preputial clefts and urethral openings located toward the base of the genital tubercle, similar to hypospadias in humans. Black circles indicate the mean likelihood of malformation, based on 10–15 litters per group, with 95% confidence intervals; the solid blue line is the best-fit regression model (Weibull) with the 95% confidence belt (dashed blue lines); the green curve and green shaded area indicate the lower and upper estimates of combination effects according to dose addition; and the gray curve represents combined effects predicted by using independent action.

Strikingly, a completely different picture emerged when we evaluated dysgenesis of the external genitalia in young male rats at PND16 and PND47. Administration of the mixture led to severe malformations at PND16 characterized by enlarged preputial clefts, and urethral openings located toward the base of the genital tubercle (not at its top, as normally expected), similar to hypospadias in humans. A laterally extending groove resembling a vaginal opening was often found at the base of the genital tubercle. The number of males affected by these abnormalities at PND16 was scored and expressed as likelihood for severe malformations [Figure 3; see also Supplemental Material, Table 4 (doi:10.1289/ehp.0900689.S1)]. Milder signs of genital dysgenesis—such as small cavities appearing on the inferior side of the genital tubercle, minor clefts in the preputial opening, or small genital tubercles—were not taken into account. At the lowest tested mixture dose, none of the effects classed as severe malformations were noted. At the second highest mixture dose, 26% of the animals were affected; at the highest tested mixture dose, virtually all animals (94%) showed severe malformations. Similar malformation rates for hypospadias, including exposure of the os penis and blind vaginal opening, became apparent at PND47. At the doses included in the mixture, none of the chemicals alone induced malformations, with the exception of vinclozolin at 50 mg/kg/day, where a low frequency was observed [see Supplemental Material, Table 4 (doi:10.1289/ehp.0900689.S1)].

Based on the dose–response data for the individual chemicals [pooled data from all studies (Table 1) and tests conducted concurrently with the mixture experiment], we calculated the probability for malformations at PND16 according to dose addition and independent action. We found that both of these concepts underestimated the observed effects by a considerable margin (Figure 3). Because DEHP and prochloraz on their own were without detectable effects at doses between 3 and 30 mg/kg/day and 5 and 50 mg/kg/day, respectively, we based the calculation of combination effects according to dose addition on two conjectures: We assumed either that DEHP and prochloraz did not contribute to the overall combination effect, or that their individual effects approached 100% as the doses increased beyond the tested range. These two assumptions define the extremes of the dose addition prediction area shown in Figure 3. The independent action prediction yielded a curve that was congruent with the high-dose extreme of the dose addition prediction area. Malformations occurred at 3-fold lower mixture doses than predicted by both of these models (Figure 3; Table 2). The steepness of the underlying dose–response curve indicates that a dose anticipated as being without effect produced malformations in 94% of the animals. Evaluated in relation to the low-dose margin of the dose-addition prediction area, the mixture caused malformations at 2-fold lower doses. The experimentally observed responses clearly exceeded the predictions, suggesting that the combined effect of DEHP, vinclozolin, prochloraz, and finasteride is synergistic with respect to genital malformations.

### Combination effects at doses around NOAELs

**Figure 4:**
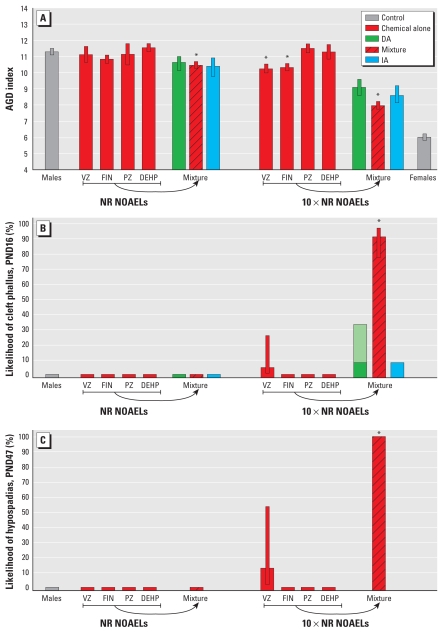
Combination effects in male rats after gestational exposure to low doses of vinclozolin (VZ; 5 mg/kg/day), finasteride (FIN; 0.01 mg/kg/day), prochloraz (PZ; 5 mg/kg/day), and DEHP (3 mg/kg/day). Doses NR NOAELs (left) and 10× NR NOAELs (right). See “Materials and Methods” for details on predicted values for dose addition (DA) and independent action (IA). Male offspring were evaluated for changes in AGD (*A*) and genital malformations at PND16 (cleft phallus; *B*) and at PND47 (hypospadias; *C*). Error bars are 95% confidence intervals. The light green part of the bar in *B* indicates the lower and upper estimates of combination effects according to dose addition. *Statistically significant compared with controls.

Next, we tested the idea that mixture effects should not occur when mixed-mode chemicals are combined at dose levels close to those used as points of departure in regulatory toxicology for the estimation of safe human exposures, such as NOAELs. DEHP, vinclozolin, prochloraz, and finasteride were combined at doses equal to their individual NR NOAELs for changes in NR that were estimated in studies preceding the mixture experiment. At these doses, none of the individual chemicals on their own produced changes in AGD, yet the combination induced a statistically significant effect, well predicted by dose addition and independent action. At 10-fold higher doses, the effects of vinclozolin and finasteride alone (but not DEHP and prochloraz) reached statistical significance. At this point, the combination strongly exacerbated the response, with AGD halfway between those expected in normal males and normal females (Figure 4A).

With weight changes of the prostate and LABC as the end points for assessment, the NR NOAEL combination did not produce effects distinguishable from untreated controls. When tested in parallel with the mixture, finasteride on its own yielded statistically significant responses for NR at its previously estimated NR NOAEL of 0.01 mg/kg/day. This prevented us from conducting the above analyses in relation to retained nipples.

Combined at their NR NOAELs, the four antiandrogens did not provoke appreciably elevated malformation rates, but the 5-fold higher mixture dose induced malformations in approximately 25% of the males. At this point, none of the single chemicals alone is likely to have led to observable malformations [Figure 3; see also Supplemental Material, Table 4 (doi:10.1289/ehp.0900689.S1)]. Even at the levels present in the 10-fold higher mixture dose, three of the single chemicals alone did not induce discernable effects, but vinclozolin on its own caused malformations in about 5% of the affected males. Yet, when combined at the doses equivalent to 10-fold NR NOAELs, we observed malformations in nearly all male offspring (Figure 4B). Similar results became apparent when malformations were analyzed later in the rats lives, at PND47 (Figure 4C).

## Discussion

In relation to certain sensitive hallmarks of disrupted male sexual differentiation, dose addition and independent action proved to be equally useful tools for the prediction of combination effects of mixed-mode antiandrogens. For alterations in AGD, retained nipples, and weight changes in prostate and LABC, reasonable anticipations of mixture effects could be achieved on the basis of dose–response descriptors for the individual mixture components. That this occurred despite the different mechanistic premises that underlie the two prediction concepts is coincidental. It is explained not by toxicologic features but by the mathematical algorithms behind each concept (Drescher and Bödeker 1995). Our results hold the promise that predictions of combination effects could be obtained in the future without conducting mixture experiments but by employing modeling approaches. Considering the high cost and long duration of reproductive toxicity studies, this might greatly aid efforts in regulatory toxicology.

All the more remarkable are our observations of synergistic interactions with genital malformations such as hypospadias. Because the affected males derived from dams that were dosed in the same way as those where additive effects on characteristics of disrupted sexual development were found, dosing errors cannot account for the deviations from expected additivity. Assessment errors introduced during the scoring of the malformations, leading to the detection of false positives, are also not a likely explanation, because we disregarded the milder forms of malformations that may be difficult to diagnose. The fact that finasteride, when tested concurrently with the mixture, proved to be more effective in inducing retained nipples than seen previously also does not explain the synergism. We found no changes in the chemicals’ effectiveness of causing malformations. As with the other end points used in this study, androgens are key factors for the normal development of the penis from the genital tubercle. The observed malformations, including hypospadias, indicate disruption of androgen action. This leads to a failure of the folding and fusion that needs to take place during development to form normal genitals. Judged from this viewpoint, the mixture would be expected to induce additive effects on malformations, too. Although we are unable to offer a mechanistic explanation for the synergisms, it seems that genital development in the rat is also governed by events that differ in important details from those operating during the regression of nipple anlagen and the formation of normal AGD. Genital tubercle development generally also includes embryonic processes requiring embryonic cell movements and apposition of the two advancing tissues (Gupta and Goldman 1986).

Recent studies suggest a previously unidentified role for the progesterone receptor, possibly interacting with the AR, in disturbed genital tubercle development (Willingham et al. 2006). *In utero* exposure to natural or synthetic progesterones can increase the risk of hypospadias in male mice, and intake during pregnancy in humans has been associated with increased risks of hypospadias (Carmichael et al. 2005; Willingham et al. 2006). Prochloraz, one of the chemicals in the mixture, was able to induce increased testicular progesterone concentrations in male rat fetuses; this effect occurred at lower dose levels than those present in the mixture investigated here (Blystone et al. 2007; Laier et al. 2006; Vinggaard et al. 2005). Thus, elevated progesterone levels due to prochloraz exposure may have a role in the synergisms with severe hypospadias that we observed in our study. Generally, synergisms may be caused by toxicokinetic interactions, where because of interference with uptake or metabolism, one or several mixture components are present at levels higher than would occur if these chemicals were given individually. However, we regard toxicokinetic interactions as an unlikely explanation, because we found dose additivity for all other hallmarks of male sexual disruption, including end points assessed around or at the same developmental age as the genital malformations.

Our findings echo the observations made by Rider et al. (2008) where a mixture of butyl benzyl phthalate, di-*n*-butyl phthalate, DEHP, vinclozolin, procymidone, linuron, and prochloraz induced frequencies of hypospadias that exceeded those predicted by dose addition. As in our study, other end points that were investigated simultaneously revealed dose-additive effects. Rider et al. (2008) used historical data for the calculation of additivity expectations. Because these data were sometimes based on dosing regimens that differed from those used in the mixture experiments, there is a degree of uncertainty as to whether the excess malformations in that study represent a true synergism. Our evaluations are based on dose–response data for single chemicals and mixtures from one large study and therefore substantiate concerns about the synergistic induction of genital malformations by antiandrogens with mixed modes of action.

The effects seen in the rat are of high relevance to the human and deserve serious consideration in ongoing risk assessment efforts. Very recently, changes in AGD (Swan et al. 2005) and suppressions of androgen synthesis have been associated with antiandrogen exposure in humans (Main et al. 2006).

A point that requires careful consideration is whether additive or even synergistic effects between antiandrogens are also likely to occur at low, environmentally relevant exposure levels. Human intakes of DEHP alone are estimated to be about 1,000 times lower than used in our study (National Research Council 2008), and similar low exposures can be expected for the other chemicals that we used. It is clear that our developmental rat model would not have produced any responses if we had combined all mixture components at such low levels. However, human populations are not just exposed to four antiandrogens, but to a larger, although poorly defined number of chemicals with a potential to interfere with androgen action. Under dose addition, theory predicts that all these chemicals, even at low levels, act together to produce combined effects (Kortenkamp et al. 2007). Whether this effect will become observable in human populations depends on the number of chemicals, their levels, and the sensitivity of humans relative to the rat.

## Conclusions

Our findings contradict the widely held view (COT 2002; VKM 2008) that chemical mixtures exhibiting varied modes of action are without combination effects at levels near supposed dose thresholds, used in regulatory toxicology as points of departure for the derivation of tolerable human exposures. Were this assumption correct, we should not have observed alterations in AGD when all four chemicals were combined at their NR NOAELs. Despite the synergism that we detected, the NR NOAEL combination did not produce appreciably elevated malformation rates, but this may be due to the lower sensitivity of this end point. It is likely that a mixture composed of a larger number of antiandrogens would have produced malformations at correspondingly lower doses, approaching NR NOAELs. Our observations show that the use of NOAELs as points of departure in risk assessment may lead to underestimations of risk when exposure is to several antiandrogens with common effect outcomes, regardless of mechanism. This highlights the deficiencies of the predominant chemical-by-chemical approach in risk assessment. It is necessary to supplant current procedures by moves toward accounting for mixture effects, and our results underscore the importance of such a paradigm change.

## Figures and Tables

**Table 1:** Studies, groups, doses, and number of time-mated animals per group.

Study	Treatment groups and doses (mg/kg/day)	No. mated (no. pregnant)
Vinclozolin dose–response and prochloraz range finding	Control: 0 (vehicle)	16 (13)
	Vinclozolin: 5, 10, 20, 40, 80, or 160	8 (6–8)
	Prochloraz: 50 or 150	8 (6–8)

Finasteride and DEHP, dose response	Control: 0 (vehicle)	16 (15)
	Finasteride: 0.001, 0.01, 0.1, 1, 10, or 100^a^	8/10 (7–9)^b^
	DEHP: 10, 30, 100, 300, 600, or 900	8 (6–8)

Prochloraz and DEHP, dose response	Control: 0 (vehicle)	16 (15)
	Prochloraz: 5, 10, 25, 50, or 100	8 (5–8)
	DEHP: 3, 10, 30, or 100	16/8 (14/6–8)^c^

Mixture study of vinclozolin, finasteride, DEHP, and prochloraz, 5:0.01:3:5 ratio	Control: 0 (vehicle)	16 (13)
	Mixture: 13.01, 65.05, or 130.10	16 (11–16)
	Vinclozolin: 5^d^ or 50^e^	8 (7–8)
	Finasteride: 0.01^d^ or 0.1^e^	8 (6)
	DEHP: 3,^d^ 15,^f^ or 30^e^	8 (5–7)
	Prochloraz: 5,^d^ 25,^f^ or 50^e^	8 (6–7)

a Dose induced perinatal death and low pup weight and had to be decreased to 50 mg/kg bw/day from PNDs 1–3 (block 1), from GD18 (block 2), and from GD11 (block 3); in block 4 the dams received 50 mg/kg bw/day during the whole dosing period. We used 50 mg/kg bw/day for data analysis.

b The finasteride dose group had 10 time-mated animals, whereas all other dose groups had 8.

c Because the 3-mg/kg DEHP dose group was not investigated in other studies, 16 rats were mated, compared with 8 in other dose groups.

d Dose of single chemical present in the 13.01-mg/kg bw/day mixture dose.

e Dose of the single chemical included in the 130.10-mg/kg mixture dose.

f Dose of the single chemical included in the 65.05-mg/kg mixture dose.

**Table 2:** Statistical uncertainty of predicted and observed effect doses for the mixture of DEHP, vinclozolin, prochloraz, and finasteride.

	Effect doses for the mixture (mg/kg/day)		
	Observed^a^	Predicted by DA	Predicted by IA
Effect level	Mean (95% CI)	Mean (95% CI)	Mean (95% CI)
Relative AGD index			
90%	11.8 (4.3–34.7)	12.2 (0.3–53.8)	12.3 (0.2–58.2)
60%	77.1 (59.4–99.8)	195.6 (147.2–262.3)*	214.3 (179.2–250.6)*

No. of nipples/areolas			
3	10.6 (6.5–15.3)	8.8 (5.6–13.6)	6.7 (4.1–13.5)
6	23.3 (17.0–29.4)	25.8 (16.8–35.7)	25.1 (16.8–41.4)

Organ weights in relation to the controls			
Ventral prostate (mg)			
90%	44.7 (13.9–79.3)	44.6 (6.2–65.2)	24.4 (0.1–68.6)
60%	103.7 (78.9–130.5	162.1 (128.6–226.5)	148.4 (98.4–200.8)
LABC (mg)			
90%	51.0 (19.3–81.9)	48.1 (3.5–78.3)	25.9 (0.02–93.1)
60%	118.9 (93.9–152.1)	179.7 (142.8–325.3)	176.2 (109.1–252.8)
Likelihood for malformation at PND16 (cleft phallus)			
10%	25.5 (21.0–31.2)	96.5–133.1^b^*	140.0–140.1^b^*
50%	37.5 (32.6–44.6)	149.1–226.2^b^*	226.2–226.4^b^*

Abbreviations: DA, dose addition; IA, independent action. Predicted effect doses are based on pooled data from studies for vinclozolin, prochloraz, finasteride, and DEHP and were calculated from the respective dose–response functions given in Supplemental Material, Table 2 (doi:10.1289/ehp.0900689.S1).

a Effect doses as calculated from the dose–response functions given in Supplemental Material, Table 2 (doi:10.1289/ehp.0900689.S1).

b No pups with malformations were observed for the tested doses of DEHP and prochloraz. For this reason, the calculation of expected mixture effect was based on two conjectures: For doses exceeding the tested dose range, we assumed either that effects were absent or that they reached maximal response. These two worst-case extrapolations define the only possible range of effects and were used to calculate the predicted effect doses for the mixture.

* Statistically significant compared with observed effect doses.
